# The relationship between implicit motives and physical activity: a scoping review

**DOI:** 10.1186/s13643-024-02678-y

**Published:** 2024-10-18

**Authors:** Julian Brummer, Nikkil Sudharsanan, Martin G. Köllner

**Affiliations:** 1 Department of Health and Sport Sciences, TUM School of Medicine and Health, Technical University of Munich, Munich, Germany; 2 Division of Physical Activity, Prevention and Cancer, German Cancer Research Center (DKFZ), Heidelberg, Germany; 3 Medical Faculty, Heidelberg University, Heidelberg, Germany; 4 Heidelberg Institute of Global Health, Heidelberg University, Heidelberg, Germany; 5 School of Psychology, SRH University of Applied Sciences Heidelberg, Heidelberg, Germany

**Keywords:** Physical activity, Exercise, Sports, Implicit motives, Achievement motive, Affiliation motive, Power motive, Affect

## Abstract

**Background:**

Interventions that leverage implicit motives — affect-based, non-conscious motivational dispositions — may increase physical activity by making physical activity more pleasurable. However, there is no evidence synthesis of the empirical data linking the major implicit motives (achievement, affiliation, and power motives) and physical activity. We aimed to close this research gap.

**Methods:**

Following a systematic literature search in the PsycInfo, PubMed, and Web of Science databases until August 2024, we performed a scoping review. We included German- or English-language publications in peer-reviewed journals or books that followed an observational or intervention study design. Studies had to link ≥ 1 major implicit motive measured via a well-established method to physical activity behavior. We critically appraised the methodological quality of the included studies using an adaptation of the JBI critical appraisal checklist for analytical cross-sectional studies and synthesized the evidence qualitatively.

**Results:**

Out of 1047 potentially relevant records, five publications (seven studies, *N* = 550) were included. All eligible studies were observational (six cross-sectional, one prospective longitudinal). The achievement motive was researched the most. The data indicated a relatively consistent positive association between physical activity and the achievement motive, particularly in athletes and in sports-specific settings. The associations with the affiliation and power motives were more mixed. Most studies were conducted in sports-specific settings. All studies elicited methodological concerns, to varying degrees.

**Conclusions:**

The available data indicate a positive association between achievement motive strength and physical activity. However, important limitations, especially the lack of intervention studies and the use of non-gold standard assessment methods, limit the confidence in the findings. More, methodologically sound research is needed to better understand the link between implicit motives and physical activity, especially in the general population.

**Systematic review registration:**

PROSPERO CRD42023392198.

## Background

Physical inactivity is widespread. The results of a recent meta-analysis indicate that only about 17% of adults meet combined aerobic and muscle-strengthening physical activity guidelines [1]. This high level of physical inactivity not only harms people’s individual health by increasing the risk of diseases like cardiovascular disease and various cancers [2–4] but also causes dramatic costs to healthcare systems [5–7]. Thus, designing and implementing effective physical activity promotion interventions is of utmost importance to counteract the negative consequences of physical inactivity. In order to design effective interventions and increase physical activity, knowledge of the causal determinants of physical activity behavior is necessary. Implicit motives might be a causal determinant of physical activity.

Implicit motives are non-conscious and affect-based motivational dispositions that lead to individuals experiencing certain activities and contexts as rewarding (i.e., *incentives*) or aversive (i.e., *disincentives*), respectively [8]. The most commonly studied implicit motives are the needs for achievement, affiliation, and power [8]. Individuals with a strong achievement motive derive satisfaction from autonomously mastering challenging tasks [8]. Those with a pronounced affiliation motive find establishing, maintaining, or restoring positive interpersonal relationships rewarding [8]. Those with a strong power motive derive satisfaction from having impact (e.g., physical, emotional) on others [8]. Implicit motives have remarkable predictive validity for activities ranging from everyday choices and preferences (e.g., a preference for feedback while engaging with challenging tasks by achievement-motivated persons) to political action (e.g., prediction of peaceful resolution of crises by the affiliation motive or initiation of armed conflicts by the power motive) [8].

People differ in their implicit motives, which can be understood as a part of human personality [9]. Implicit motives appear to be conceptually and empirically different from related concepts such as explicit (i.e., conscious) motives [8, 10] or the Big-Five personality traits [11].^1^

**Fig. 1:**
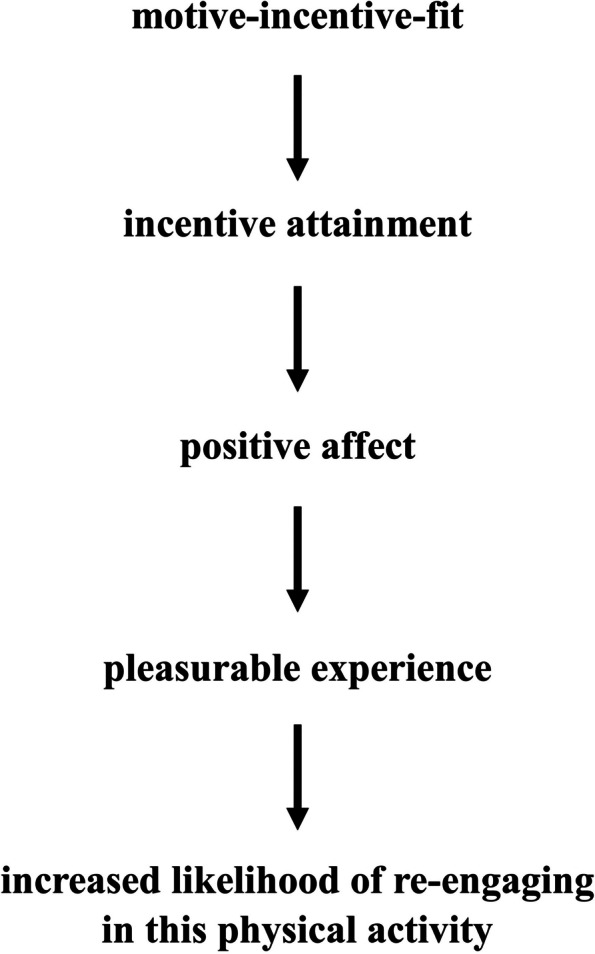
The proposed mechanism of how implicit motives might causally influence physical activity behavior. Note: If the incentives provided by a physical activity context fit a person’s implicit motives, incentive attainment is facilitated. Incentive attainment, in turn, leads to positive affect. Experiencing positive affect in the context of being physically active should make the overall physical activity experience pleasurable, thereby increasing the likelihood of engaging in that physical activity behavior again in the future. Thus, in essence, if a person’s implicit motives can be satisfied by a given physical activity context, the physical activity should be perceived as more affectively rewarding and pleasurable, thus motivating future physical activity in this context. Figure available at https://osf.io/8jyhg/ [12], under a CC-BY4.0 license

Their hedonic basis is why implicit motives might be a causal determinant of physical activity behavior: If a person’s implicit motives and the incentives provided by the context of being physically active align (i.e., a motive-incentive-fit exists) and, thus, the person can successfully attain motive-specific incentives (i.e., the implicit motives get satisfied), positive affect should be the consequence [13]. Persons with stronger implicit motives should accordingly also have a greater capacity to derive positive affect from the satisfaction of their implicit motives [8, 13]. At this stage, the concepts of hedonism [14] and motive-driven learning [13] become relevant: Through their effects on affect, implicit motives play a key role in the learning of stimuli, behaviors, and contexts that are related to the affective experience [13]. In the context of physical activity, this could mean that the positive affect resulting from encountering a motive-incentive fit and attaining motive-specific incentives would contribute to *learning* that this physical activity context is one of reward [13]. Simply put, the positive affect resulting from the respective physical activity behavior/context should serve as a “motivating force,” thereby increasing the probability of re-engaging in that physical activity behavior again in the future. Figure 1 summarizes this proposed mechanism.

For example, highly achievement-motivated persons might enjoy a physical activity context which allows them to get better at a certain physical activity, and thus beneficially respond to environments where they are provided with personalized feedback [10]. Highly affiliation-motivated individuals might be particularly responsive to physical activity contexts that include familiar social environments and cooperation [10]. Particularly power-motivated persons might be drawn to physical activity contexts in which they can guide and support others or engage in direct competition with others [10].

Given that individuals with stronger implicit motives consequently have a greater capacity to derive positive feelings from implicit motive satisfaction [8, 13], a positive association between implicit motives and physical activity would be expected *if* motive-specific incentives are available that initiate the proposed cascade.

Despite a plausible theoretical underpinning, prior reviews neglected implicit motives in the context of physical activity behavior. Yet, the apparent uniqueness of the implicit motive concept also means that implicit motives might have incremental utility for understanding, predicting, or promoting physical activity behavior. For instance, knowing how implicit motives are related to physical activity could inform the design of interventions that increase physical activity by creating opportunities for individuals to satisfy their dominant implicit motives.

In order to provide a systematic overview of the breadth of evidence on the relationship between implicit motives and physical activity, and identify important research gaps that should be addressed in the future [15, 16], we conducted a scoping review of the empirical data linking physical activity and the three major implicit motives. Following a systematic literature search, we aimed to answer the following research questions:

1. What is the relationship between implicit motives and physical activity (based on both observational and intervention studies)?
2. Have studies tried to tailor a physical activity promotion intervention based on persons’ implicit motives (based on intervention studies)?
  2.1. If yes, did these tailored interventions lead to larger effects compared to other tailored or non-tailored interventions (based on intervention studies)?

### Methods

This scoping review was registered with PROSPERO (CRD42023392198) and its reporting follows the PRISMA extension for scoping reviews (PRISMA-ScR) guideline [17].^2^

A comprehensive literature search was undertaken in the PsycInfo, PubMed, and Web of Science databases. The main search was performed on January, 23 (PubMed, Web of Science) and January, 24 (PsycInfo), 2023, respectively. A supplementary search in the same three databases was performed on August 07, 2024. The search string combined keywords capturing implicit motives and different forms of physical activity. As an example, the search string used in the Web of Science database was: TS = ((implicit AND (motiv* OR achievement OR affiliation OR affiliation-intimacy OR power)) AND (exercis* OR sport$ OR “physical activity”)).

JB conducted the database searches. JB imported the records into a reference manager (main search: Zotero, supplementary search: EndNote) to manage the screening process and remove duplicates. JB screened the titles and abstracts against the eligibility criteria. NS cross-checked a subset of records for eligibility in this stage of the screening process (main search: 200 [26.7%] randomly selected records; supplementary search: 26 [100.0%] records). Discrepancies were resolved by discussion and ineligible texts were discarded. In the next step, the full texts of the remaining English-language publications were screened for eligibility by JB and NS; German-language texts were only screened by JB. Discrepancies were resolved by discussion. Ineligible texts were discarded. The reference lists of eligible publications were hand-searched by JB to find any additional relevant literature; thereby identified full-texts were screened for eligibility by JB.

We included publications if the following inclusion criteria were met: (1) written in English or German and published prior to our search dates; (2) original research published in peer-reviewed scientific journals or books^3^; (3) observational or intervention study design, whereby intervention studies did not have to be randomized controlled trials (e.g., one-arm pilot interventions were eligible, too); (4) implicit motives measured via the gold standard measurement Picture Story Exercise [18], the Thematic Apperception Test in the tradition of Heckhausen [19], the Implicit Association Test by Slabbinck et al. [20], the Multi Motive Grid [21], or the Operant Motive Test [22] were linked to physical activity behavior (e.g., minutes of physical activity/week).

We excluded publications if at least one of the following exclusion criteria was met: (1) wrong language; (2) wrong document type (e.g., conference papers); (3) implicit motives not measured or assessed with an ineligible method; (4) physical activity not measured or only ineligible aspects of physical activity assessed (e.g., motor performance, intentions); (5) no individual-level data. No restrictions regarding publication date or participant age were applied. Relevant information were extracted from the publications by JB and independently double-checked by NS and MGK. The following information were extracted: (1) authors and publication year; (2) study design; (3) sample characteristics (sample size and if available age and sex); (4) short study summary; (5) methods used to assess implicit motives and physical activity and, if applicable, other relevant psychological concepts; (6) main results, based on reported correlation and/or regression coefficients and comparable results.

In order to also systematically generate insights into the methodological quality of included studies, JB and MGK independently conducted a critical appraisal using an adaptation of the JBI critical appraisal checklist for analytical cross-sectional studies [23]. Subsequently, the two authors formed a consensus rating for each included study by discussing their individual ratings.

We synthesized the evidence qualitatively.

## Results

**Fig. 2:**
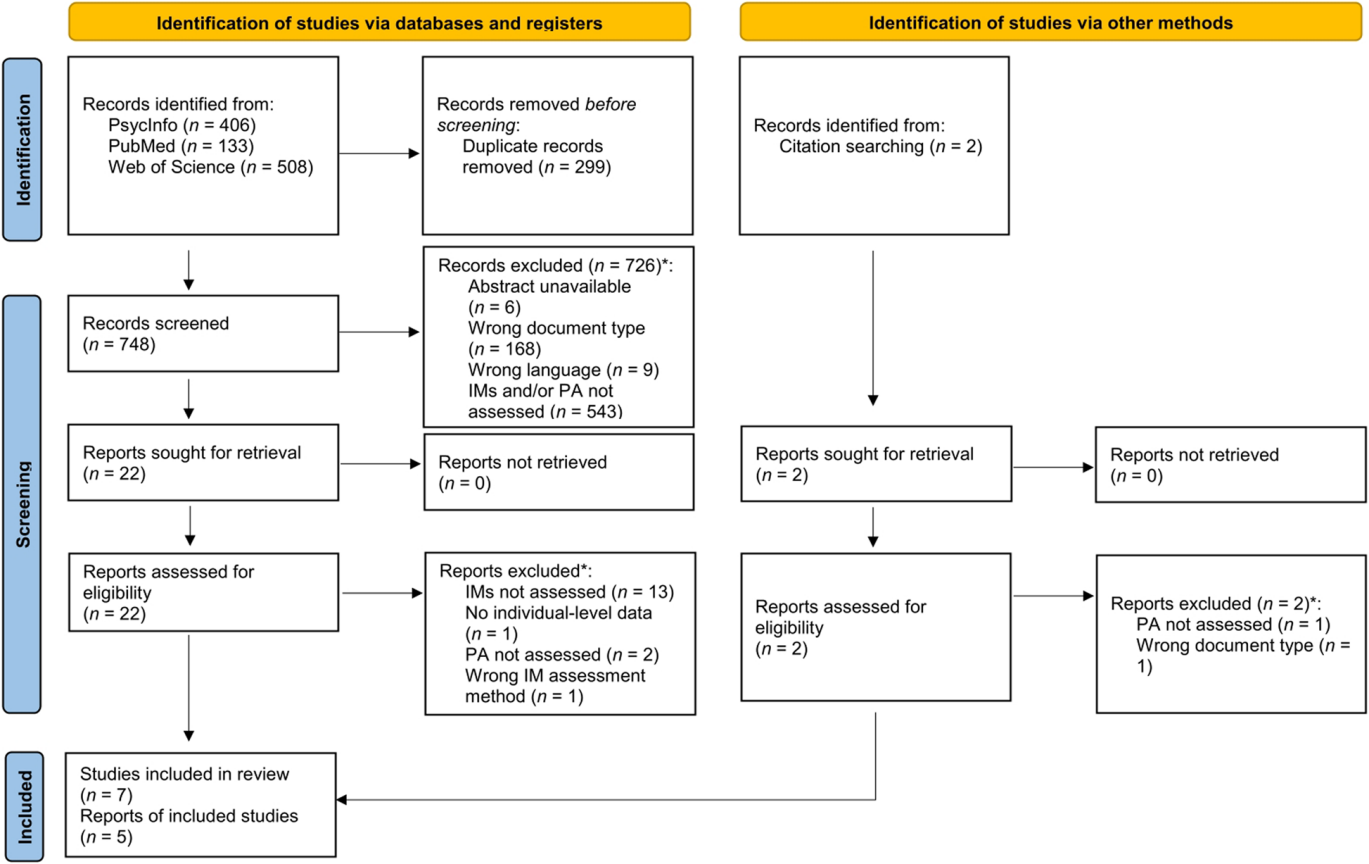
PRISMA flow diagram. Note: Depicted are the processes of literature search, screening, and selection using the PRISMA flow diagram template [29]. The asterisk indicates that there may have been multiple reasons for excluding any single publication; here, only one reason is counted per publication. Abbreviations: IMs = implicit motives; PA = physical activity. Figure available at https://osf.io/8jyhg/ [12], under a CC-BY4.0 license

The literature search identified 1047 potentially relevant records. Ultimately, five eligible publications reporting seven unique studies and comprising a total of 550 participants were identified [24–28]. Figure 2 shows a PRISMA flow diagram [29].

Three publications reported the results of one study each [25–27]. Gröpel et al. [24] reported findings from three studies and Wegner et al. [28] reported findings from two studies. However, Study 3 reported by Gröpel et al. [24] and Study 1 reported by Wegner et al. [28] used data from the same sample but focused on different implicit motives.

All eligible studies had an observational design. Four publications [24, 25, 27, 28] reported findings from six cross-sectional studies; one publication [26] reported findings from a prospective longitudinal study. No eligible intervention study was identified (research question 2); thus, research question 2.1 could not be answered.

Table 1 provides a detailed summary of the included publications.

**Table 1:** Summary of relevant studies

Authors, year	Study design and summary	Sample size and characteristics	Measurement of IMs and PA	Main results
Gröpel et al. [24]	Cross-sectional: Three studies with samples of different expertise levels investigated the hypotheses that (a) both the implicit achievement motive as well as the explicit achievement motive are positively associated with sport participation and (b) that the congruence between a strong implicit achievement motive and a strong explicit achievement motive predicts sport participation	Study 1: *n* = 112 (88f, 24 m) college students enrolled in a university leisure sports program; age: *M* = 20.0 years (*SD* = 3.8)	Study 1: IMs: MMG (hope of success score calculated by adding up the respective scores for 14 pictures); PA: number of days/week a participant engaged in sports activities/practice via a single-item self-report	Study 1: The implicit achievement motive scores ranged from 0 to 12 (*M* = 6.94, *SD* = 2.38, Cronbach’s Alpha = 0.65). Sports participation: *M* = 2.78, *SD* = 1.67 days per week Statistically significant, positive, small-to-moderate correlation between the implicit achievement motive and PA (*r* = 0.23). Statistically significant positive main effect of the implicit achievement motive in regression analyses (*β* = 0.23–0.25). The interaction between the implicit achievement motive and the explicit achievement motive was statistically unrelated to sports participation
		Study 2: *n* = 63 (31f, 32 m) amateur athletes from several sport clubs and kinds of sports (e.g., tennis, triathlon, basketball); age: *M* = 28.7 years (*SD* = 10.3)	Study 2: IMs: six-picture PSE (regression was used to correct the raw motive scores for protocol length); PA: number of days/week a participant engaged in sports activities/practice via a single-item self-report Other relevant psychological variables assessed: explicit achievement motive (inferred from participants’ personal goals)	Study 2: No descriptive statistics for the implicit achievement motive. Sports participation: *M* = 4.44, *SD* = 1.23 days per week Statistically significant, positive, moderate-size correlation between the implicit achievement motive and PA (*r* = 0.33). Statistically significant positive main effect of the implicit achievement motive in regression analyses (*β* = 0.32–0.33). The interaction between the implicit achievement motive and the explicit achievement motive was statistically unrelated to sports participation
		Study 3: *n* = 30 (0f, 30 m) elite tennis players; age: *M* = 29.9 years (*SD* = 4.5); Notably, this was the same study sample as in Study 1 reported by Wegner et al. [28]	Study 3: IMs: OMT PA: PA: number of hours/week a participant engaged in sports activities/practice via a single-item self-report Other relevant psychological variables assessed: explicit achievement motive (achievement scale of the PRF)	Study 3: The implicit achievement motive scores ranged from 1 to 5 (*M* = 2.79, *SD* = 1.13). Sports participation (practice time): *M* = 11.60, *SD* = 9.80 h per week Statistically significant, positive, large correlation between the implicit achievement motive and PA (*r* = 0.58). Regression analyses controlled for participants’ age because there was a statistically significant, large, negative correlation with practice hours. Statistically significant positive main effect of the implicit achievement motive in regression analyses (*β* = 0.50–0.53). The interaction between the implicit achievement motive and the explicit achievement motive was statistically unrelated to sports participation. Controlling for participants’ sports experience (years of playing) and ATP ranking did not significantly affect any of these regression results
Jacob and Guarnaccia [25]	Cross-sectional; This study assessed life satisfaction and its motivational and behavioral correlates in an elderly sample. For this, among other measures, participants’ implicit achievement motive and implicit affiliation motive were assessed and data on their exercise behavior collected	*n* = 97 (75f, 22 m); age: *M* = 74.7 years (*SD* = 6.5)	IMs: five-picture PSE, but the stories were not written down by the participants directly; rather, they orally told stories, which were taped and later transcribed PA: no details are provided on how exercise behavior was assessed, except for that it was self-reported Other relevant psychological variables assessed: explicit achievement and affiliation motives (PRF)	Descriptive statistics for the implicit achievement motive and the implicit affiliation motive are not reported herein due to conflicting information in the original publication, but the authors describe their mean values as low. No descriptive statistics for PA Intercorrelation between the implicit achievement motive and PA was statistically non-significant, positive, and small (*r* = 0.07). Intercorrelation between the implicit affiliation motive and PA was statistically non-significant, positive, and small-to-moderate in size (*r* = 0.19). Statistical significance was set at *p* = 0.01
Kopp et al. [26]	Prospective longitudinal; This multicenter study investigated whether the implicit achievement motive, the explicit achievement motive, autonomous forms of exercise motivation (intrinsic motivation, identified regulation), and controlled forms of exercise motivation (introjected regulation, external regulation) would prospectively predict gym attendance over 30 consecutive weeks in new gym members. Data were collected at four measurement points (weeks 1, 4, 15, and 30 after the start of the gym membership). While the implicit achievement motive is the focus of the investigation, the authors state that they also analyzed the relationship between gym attendance and the implicit affiliation motive and the implicit power motive, respectively	*n* = 149 completed the entire study; at baseline 138f, 91 m, age: *M* = 32.1 years (*SD* = 12.4)	IMs: computer-based five-picture PSE (regression was used to correct motive scores for protocol length) PA: self-reported gym attendance (average days per week since the last survey) was assessed using a single-item self-report; actual gym attendance was assessed electronically using magnetic check-in cards Other relevant psychological variables assessed: explicit motives (Unified Motive Scales); autonomous and controlled forms of motivation (Exercise Self-Regulation Questionnaire)	Raw (uncorrected) implicit achievement motive score: *M* = 4.26, *SD* = 2.34; actual gym attendance (days/week): week 4 (*n* = 221): *M* = 1.65, *SD* = 0.87; week 15 (*n* = 196): *M* = 1.28, *SD* = 1.05; week 30 (*n* = 149): *M* = 0.99, SD = 1.02; Self-reported gym attendance (days/week): week 4: *M* = 2.44, *SD* = 1.04; week 15: *M* = 2.06, *SD* = 1.12; week 30: *M* = 1.84, *SD* = 1.26; Controlling for participants’ gender, there were only a few statistically significant correlations between the implicit achievement motive and PA: A positive, small-to-moderate-sized correlation with self-reported gym attendance at 30 weeks (*r* = 0.18) and a positive, small-to-moderate-sized correlation with actual gym attendance at 15 weeks (*r* = 0.16); all other correlations at 4, 15, and 30 weeks were not statistically significant Complex multilevel growth modeling showed that the implicit Achievement motive did not statistically significantly predict gym attendance (both self-reported and actual) over the 30-week period. Similarly, the interaction between the implicit achievement motive and the explicit achievement motive did not statistically significantly predict gym attendance (both self-reported and actual) over the 30-week period Additional analyses showed that participants who completed all four measurements had higher scores for the implicit achievement motive than participants who did not. They also had a higher actual gym attendance at 4 weeks. Both effects were small-to-moderate in size and statistically significant With regards to the implicit affiliation and power motives, no statistically significant correlations between these motives and gym attendance were found at either measurement point. In addition, the interactions between the implicit affiliation and power motives and their respective explicit counterparts did not predict gym attendance
Schütz and Schultheiss [27]	Cross-sectional; Among other research questions, this study investigated whether IMs predict sports performance, which was partly operationalized as training hours in the previous week, and whether this relationship is moderated by the presence of adequate achievement and affiliation incentives	*n* = 67 gymnasts involved in age-appropriate competitions of varying levels (38f, 29 m); age: *M* = 26.5 years (*SD* = 7.8)	IMs: computer-based, six-picture PSE (motive scores were corrected for protocol length) PA: sum of self-reported training hours (not limited to gymnastics training) for every day of the previous week Other relevant psychological variables assessed: explicit motives (PSE-Questionnaire); perceived failure (self-devised self-report item), perceived autonomy (respective sub-scale of the Psychological Need Satisfaction in Exercise Scale), training alone vs. with others (self-report item)	Raw (uncorrected) motive scores: implicit achievement motive: *M* = 7.18, *SD* = 3.73; implicit affiliation motive: *M* = 8.75, *SD* = 4.41; implicit power motive: *M* = 5.83, *SD* = 3.31; Based on *n* = 56, a statistically significant, positive, moderate-sized correlation was found between the implicit achievement motive and PA (*r* = 0.27). A statistically non-significant, negative, small correlation was found between the implicit affiliation motive and PA (*r* = − 0.07). No correlation was found between the implicit power motive and PA (*r* = 0.00) Participants’ sex, age, and athletic ability did not moderate the relationship between the implicit achievement motive and PA Results did not support the authors’ hypothesis that suitable achievement (perceived control, perceived failure) and affiliation (training together vs. alone) incentives moderated the relationship between the implicit achievement motive and the implicit affiliation motive and training hours, respectively Exploratory analyses further indicated that congruence between IMs and explicit motives was not associated with training hours. The implicit power motive was positively associated with training hours only in participants that were low in explicit power motivation but not in persons with medium or high explicit power motivation
Wegner et al. [28]	Cross-sectional; Across two studies, the relationship between elite athletes’ implicit power motive and their practice times was assessed. The focus of this investigation was on the implicit power motive’s approach/hope and avoidance/fear components, whereby it was hypothesized that the fear component of the implicit power motive is positively associated with current, maximum, and additional practice hours	Study 1: *n* = 30 elite tennis players (0f, 30 m); age: *M* = 29.9 years (*SD* = 4.5); Notably, this was the same study sample as in Study 3 reported by Gröpel et al. [24]	Study 1: IMs: OMT PA: Practice time was assessed using a single-item self-report asking for current weekly practice hours (one month after the start of the season) and at the time in the past when the player practiced most intensely (i.e., maximum practice times) Other relevant psychological variables assessed: explicit motives (PRF)	Study 1: Hope component: *M* = 8.67, *SD* = 2.28; Fear component: M = 0.57, *SD* = 0.68; For the hope component, a statistically significant, negative, large correlation was found with current practice times (*r* = − 0.50), whereas a statistically non-significant, negative, moderate-size correlation was found with the maximum practice times (*r* = − 0.33). For the fear component, statistically significant, positive, moderate-to-large correlations with current (*r* = 0.38) as well as maximum practice times (*r* = 0.36) emerged, respectively Results of multiple regression analyses showed a statistically significant, negative association between current practice times and the hope component (*β* = − 0.45) as well as a trend-level significant positive association with the fear component (*β* = 0.30). For the maximum practice times, a trend-level significant negative and positive association emerged with the hope (*β* = − 0.30) and fear components (*β* = 0.34), respectively
		Study 2: *n* = 32 elite karateka (0f, 32 m); age: *M* = 23.2 years (*SD* = 4.8)	Study 2: IMs: MMG PA: Current weekly practice times were assessed like in Study 1. Furthermore, participants were asked how much time they practice per week in addition to the regular team practice Other relevant psychological variables assessed: explicit motives (PRF)	Study 2: Hope component: *M* = 5.09, *SD* = 2.26; Fear component: *M* = 6.53, *SD* = 2.18; For the hope component, a statistically non-significant, positive, small-to-moderate correlation with current practice times was found (*r* = 0.16) and a statistically non-significant, negative, small-to-moderate correlation with additional practice times was found (*r* = − 0.19). For the fear component, a trend-level significant, positive, moderate-sized correlation with current practice times emerged (*r* = 0.32). In addition, a statistically significant, positive, moderate-to-large correlation was found with additional practice times (*r* = 0.45) Results of multiple regression analyses showed a statistically non-significant, positive association between current practice times and the hope component as well as a trend-level significant positive association with the fear component (β = 0.33). For the additional practice times, a statistically non-significant, negative and a statistically significant, positive association (β = 0.45) emerged with the hope and fear components, respectively

*IMs* Implicit motives, *M* Mean, *MMG* Multi Motive Grid, *OMT* Operant Motive Test, *PA* Physical activity, *PRF* Personality Research Form, *PSE* Picture Story Exercise, *SD* Standard deviation, *TAT* Thematic Apperception Test

### Main findings

Six studies (85.7%) investigated the relationship between the achievement motive and physical activity [24–27]. Five cross-sectional studies consistently found small-to-large, positive associations between the achievement motive and self-reported sports participation in a university leisure sports program and various sports clubs [24], self-reported exercise behavior [25], and self-reported training hours in gymnasts [27]. Most of these associations also reached statistical significance. The only prospective longitudinal study reported mixed (positive or negligible) associations between the achievement motive and self-reported and actual gym attendance across the study period [26].

Three studies (42.9%) examined the relationship between the affiliation motive and physical activity [25–27]. Two cross-sectional studies found statistically non-significant small-to-moderate, positive, and small, negative associations between the affiliation motive and self-reported exercise behavior [25] and self-reported training hours in gymnasts [27], respectively. The longitudinal study by Kopp et al. [26] found no significant associations between the affiliation motive and gym attendance.

Four studies (57.1%) investigated the relationship between the power motive and physical activity [26–28]. One cross-sectional study did not find an association between the power motive and self-reported training hours in gymnasts [27]. In two cross-sectional studies, Wegner et al. [28] found that the fear component of the power motive specifically was relatively consistently, positively associated with sports practice workloads in elite tennis players and karateka. Results for the hope component were inconsistent but pointed towards a negative association with physical activity [28]. The longitudinal study by Kopp et al. [26] found no significant associations between the power motive and gym attendance.

### Moderator analyses

A narrative review did not reveal a moderating effect of age or sex/gender. However, the associations between the achievement motive and physical activity were particularly consistent and pronounced in athletes vs. non-athletes and in sports-specific vs. non-sports-specific settings.

### Critical appraisal

All included publications elicited methodological concerns, to varying degrees. Of concern was a mostly insufficient identification and consideration of potential confounding variables [24, 25, 28]. Furthermore, only in four studies [24–27], the gold-standard implicit motive assessment method, i.e., the Picture Story Exercise, was used. All seven studies measured physical activity via self-report [24–28], often via self-devised single-item questionnaires. Only one study also electronically measured gym attendance using magnetic check-in cards [26], thereby providing objective, albeit indirect, data on physical activity.

## Discussion

The present research is the first scoping review of empirical studies linking implicit motives and physical activity. We identified five eligible publications reporting seven unique observational studies; no intervention study was identified. Achievement motive strength was relatively consistently positively associated with physical activity, especially in athletes and in sports-specific settings. There was less and more mixed evidence on the associations between physical activity and affiliation and power motives. Overall, a paucity of studies — particularly intervention studies — examining the relationship between implicit motives and physical activity, methodological shortcomings, and a focus on sports contexts/athletic populations were identified by this scoping review.

### Discussion of the findings

The achievement motive was the most-researched implicit motive. Most studies showed a positive association between physical activity and the achievement motive, i.e., persons with a stronger achievement motive tended to engage in more physical activity. This positive association between achievement motive strength and physical activity is in line with the proposed mechanism of how (the satisfaction of) implicit motives may causally impact physical activity behavior (Fig. 1): Persons with stronger implicit motives can derive stronger positive affect from the satisfaction of their implicit motives [8, 13]. Hence, a positive association between implicit motive strength and physical activity would be expected since stronger positive affect should also translate into a stronger motivation to re-engage in the respective physical activity. However, this only holds true if motive-specific incentives are present and can be attained. Since sports contexts — which were commonly studied — typically provide ample achievement incentives, such as performance feedback and the opportunity to set new personal records [10], a motive-incentive fit is likely. Moreover, through their effects on affect, implicit motives appear to play an important role in the learning of stimuli, behaviors, and contexts that are related to the affective experience [13]. This might further explain these findings.

The positive associations between the achievement motive and physical activity were more consistent and larger in athlete compared to non-athlete samples. Athletes might have learned to associate physical activity with personal standards of excellence, which is the driving force of the achievement motive. This learned association might be weaker in individuals who only participate in physical activity for health reasons.

However, these results should be interpreted with caution due to the methodological limitations of the respective studies, which limit the confidence in the findings to some degree.

Interestingly, in contrast to the achievement motive, there were no consistent (positive) associations between physical activity and the strength of the affiliation and power motives, respectively. It is possible that more research would further clarify the relationship between these implicit motives and physical activity, as only few studies — in part with important methodological limitations — investigated them. Alternatively, it is possible that the study contexts lacked appropriate incentives for affiliation (e.g., cooperative settings) and power (e.g., direct comparisons, guiding others) motives [10].

Only one publication considered the presence of motive-specific incentives [27]. Surprisingly, the authors did not find any evidence that the presence of suitable incentives for the achievement motive (perceived failure, perceived autonomy) and the affiliation motive (training with others vs. alone) moderated the respective relationship with training hours in gymnasts [27].

### Limitations and directions for future research

At present, it is not possible to infer causality; future studies will need to better control for confounding and apply other study designs to clearly elucidate the temporal order, distinguish cause and effect, and clarify the potential role of confounders.

To optimally infer the causal effect of implicit motives on physical activity, it would be necessary to randomize implicit motives. However, randomizing implicit motives directly appears infeasible, for example, because it would necessitate highly invasive interventions, such as controlling parental behavior [30]. Moreover, there are still many open questions pertaining to the stability of implicit motives and changes therein [9], rendering the idea of experimentally altering adults’ implicit motives questionable, too. Thus, a promising alternative approach could be to use implicit motives as the personalization target in tailored physical activity promotion interventions. Since people differ in their implicit motives [9], this could be leveraged to deliver intervention content that taps into persons’ dominant implicit motives. Thereby, the cascade proposed in Fig. 1 could be started deliberately, and, consequently, physical activity made more pleasurable. Whether measurable increases in physical activity would be the consequence remains to be determined.

In any case, such a study would hold great value from multiple perspectives. First, it would generate important insights into the practical value of implicit motives. Since a large part of the empirical literature surrounding implicit motives and their relevance for behavioral outcomes is observational in nature [31], a randomized controlled trial could strengthen the available database. As the proposed mechanism depicted in Fig. 1 is consistent with the theory surrounding implicit motives, a randomized controlled trial whose design adheres to this theory could help to further develop the theory surrounding implicit motives — either by supporting current theorizing or by refuting it. Specifically, a randomized controlled trial would help to clarify the temporal order of events (i.e., implicit motives preceding behavior) and eliminate, or at least strongly limit, confounding. In addition, such a study would uniquely add to the field of personalized behavior change interventions — in this case, personalized physical activity promotion [32, 33]. Lastly, if the personalization based on implicit motives turns out to indeed result in clinically meaningful increases in PA, the “toolbox” for physical activity promotion could be expanded.

Furthermore, as mentioned above, methodological limitations limit the confidence in the implications of the current body of evidence. Hence, we believe it to be important that future studies adopt measures that increase their methodological quality. This should include prioritizing standardized versions of the Picture Story Exercise for assessing implicit motives [8, 34]. Similarly, direct and objective measures of physical activity (e.g., accelerometry) should be deployed more, especially when assessing overall physical activity as compared to sports-/exercise-specific physical activity. Alternatively and/or complementarily, validated physical activity questionnaires should be utilized. When conducting further observational studies, it will also be important to put greater emphasis on identifying and controlling for potential confounding variables. Collectively, these measures will increase the methodological quality of future studies, and thus the confidence in their results.

It will also be worthwhile to conduct studies that focus on more diverse, yet specific populations (e.g., different age groups); different types of physical activity (e.g., aerobic vs. muscle-strengthening activities); and on specific implicit motives. Once more studies have been conducted on the relationship between implicit motives and physical activity, it will certainly also be worthwhile to perform a meta-analysis of published results. In the present work, we decided not to perform a meta-analysis because of the small overall body of literature and the pronounced heterogeneity in the methodology of the included studies.

Lastly, we cannot exclude the possibility of publication bias leading, potentially leading to an overestimation of the relationship between implicit motives and physical activity. Future meta-analyses should thus also incorporate an investigation of publication bias (e.g., using funnel plots).

## Conclusion

The results of this scoping review suggest that the strength of the implicit achievement motive is positively associated with physical activity, particularly in athletes and in sports-specific settings. This scoping review further identified the need for more and methodologically optimized studies to improve our understanding of the relationship between implicit motives and physical activity, particularly among the general population.

## Data Availability

Data sharing is not applicable to this article as no datasets were generated or analyzed during the current study. All relevant information are included in the main article and the online supplementary materials, which are available at https://osf.io/8jyhg/ [12].

## Footnotes

1 The following supplementary information can be found in the Online Supplementary Materials [12]: further details on implicit motives (concept; measurement; distinctions), methodological details (search strategy; critical appraisal; data synthesis), more fine-grained results (summary of an additional publication, which was ineligible but extraordinarily thematically fitting; subgroup and sensitivity analyses; results of the critical appraisal); further points of discussion (randomizing and altering implicit motives); and deviations from the PROSPERO protocol with the according rationale.

2 Some additional methodological details included in the PRISMA-ScR guideline can be found in the Online Supplementary Materials [12].

3 We did not search for (publications in) books specifically; however, if we had found an otherwise eligible (publication in a) book via one of the searched databases, we would have included it.
